# P047 Scalp necrosis: a rare manifestation of giant cell arteritis

**DOI:** 10.1093/rap/rkag113.077

**Published:** 2026-11-03

**Authors:** James Keasley, Kirsty Levasseur

**Affiliations:** Russells Hall Hospital, Dudley, United Kingdom; Russells Hall Hospital, Dudley, United Kingdom

## Abstract

**Introduction:**

Cutaneous manifestations of giant cell arteritis (GCA) are considered to be rare. There is limited evidence on scalp necrosis and its treatment in the context of GCA. Current literature only describes case reports. Reports suggest that cutaneous manifestations are often associated with later presentations and other ischaemic phenomena such as visual disturbance.

**Case description:**

In June 2025, a 75-year-old female patient attended with a several month history of pain in her jaw when eating and occasionally when talking. She had a background of vitamin B12 deficiency, previous humeral head fracture, left total knee replacement and laparoscopic cholecystectomy. She had had several falls in the weeks prior to diagnosis that were put down to her struggling to get in and out of a chair. She had had shoulder and hip girdle stiffness for several months. Two weeks prior to presentation, she had bilateral temporal headaches, jaw claudication and modest weight loss. She had also had several episodes of short-lived horizontal diplopia but ophthalmology review did not yield any specific concerns. In the preceding months, her family noted slight cognitive decline. She did not have limb claudication, fevers or night sweats.

On initial rheumatology review, we noted a 2x3cm right-sided erythematous ulcer on her scalp. This had progressed in the 2 weeks prior to presentation. There were no bruits, temporal artery pulsations were not palpable, and the temporal areas were tender. She did not have any cranial nerve palsies.

She had raised inflammatory markers with a CRP of 51 and ESR of 68. Her haemoglobin was 119 and her white cell count was 12.4. Temporal artery ultrasound demonstrated bilateral halo sign but normal axillary vessels.

She commenced 60 mg of prednisolone, lansoprazole, calcium carbonate and colecalciferol, and alendronate. We requested an MRI head which only demonstrated chronic ischaemic foci but no other concerning vascular phenomena. Within 2 weeks, the lesion had healed over, and the rest of her symptoms, including claudication, double vision and stiffness, had improved. Her GP had started topical antibiotics for the ulcerated area and made a referral to the Dermatology team, without making the potential connection to GCA.

**Discussion:**

Evidence on the cutaneous manifestations of GCA is limited, but they are thought to be rare. As scalp necrosis is felt to be an ischaemic complication, it would make sense for this to be a reasonably late presentation. This was the case for this patient, who had had symptoms for several months. While this lesion was reasonably small, scalp necrosis can be very extensive. This lesion was steroid responsive. Limited reviews of collected case reports suggest that scalp necrosis can be associated with poor prognosis, including mortality and association with irreversible visual loss. It is possible that GCA causing scalp necrosis could present to Dermatology, and so they should also be aware of the problem.

**Key learning points:**

1. While scalp necrosis is rare, it is an important manifestation of GCA to recognise.

2. Depending on the size of the lesion, support from the multidisciplinary team may be beneficial for optimal management.

3. Scalp necrosis can be associated with worse clinical outcomes.

